# Rapid screening for chromosomal aneuploidies using array-MLPA

**DOI:** 10.1186/1471-2350-12-68

**Published:** 2011-05-17

**Authors:** Jing-Bin Yan, Miao Xu, Can Xiong, Da-Wen Zhou, Zhao-Rui Ren, Ying Huang, Monique Mommersteeg, Rinie van Beuningen, Ying-Tai Wang, Shi-Xiu Liao, Fanyi Zeng, Ying Wu, Yi-Tao Zeng

**Affiliations:** 1 Institute of Medical Genetics, Children's Hospital of Shanghai, Shanghai Jiao Tong University, Shanghai, P.R. China; 2 Key Lab of Embryo Molecular Biology, Ministry of Health, and Shanghai Lab of Embryo and Reproduction Engineering, Shanghai, P.R. China; 3 PamGene International BV, 's-Hertogenbosch, The Netherlands; 4 Medical Genetic Institute of Henan Province, the People's Hospital of Henan Province, Zhengzhou, P.R. China; 5 Institute of Medical Sciences, Shanghai Jiao Tong University School of Medicine, Shanghai, P.R. China

## Abstract

**Background:**

Chromosome abnormalities, especially trisomy of chromosome 21, 13, or 18 as well as sex chromosome aneuploidy, are a well-established cause of pregnancy loss. Cultured cell karyotype analysis and FISH have been considered reliable detectors of fetal abnormality. However, results are usually not available for 3-4 days or more. Multiplex ligation-dependent probe amplification (MLPA) has emerged as an alternative rapid technique for detection of chromosome aneuploidies. However, conventional MLPA does not allow for relative quantification of more than 50 different target sequences in one reaction and does not detect mosaic trisomy. A multiplexed MLPA with more sensitive detection would be useful for fetal genetic screening.

**Methods:**

We developed a method of array-based MLPA to rapidly screen for common aneuploidies. We designed 116 universal tag-probes covering chromosomes 13, 18, 21, X, and Y, and 8 control autosomal genes. We performed MLPA and hybridized the products on a 4-well flow-through microarray system. We determined chromosome copy numbers by analyzing the relative signals of the chromosome-specific probes.

**Results:**

In a blind study of 161 peripheral blood and 12 amniotic fluid samples previously karyotyped, 169 of 173 (97.7%) including all the amniotic fluid samples were correctly identified by array-MLPA. Furthermore, we detected two chromosome X monosomy mosaic cases in which the mosaism rates estimated by array-MLPA were basically consistent with the results from karyotyping. Additionally, we identified five Y chromosome abnormalities in which G-banding could not distinguish their origins for four of the five cases.

**Conclusions:**

Our study demonstrates the successful application and strong potential of array-MLPA in clinical diagnosis and prenatal testing for rapid and sensitive chromosomal aneuploidy screening. Furthermore, we have developed a simple and rapid procedure for screening copy numbers on chromosomes 13, 18, 21, X, and Y using array-MLPA.

## Background

Chromosome abnormalities are a well-established cause of pregnancy loss. The most common are autosomal aneuploidy (~75%), followed by polyploidy (~13%), sex chromosome abnormalities (~8%) and structural imbalance (~4%) [1,2]. Trisomy of chromosome 21, 13, or 18 as well as sex chromosome aneuploidy account for 60-80% of abnormal fetal karyotypes detected in cultured amniotic fluid cells [3].

For non-mosaic standard trisomy, cultured karyotype analysis has been considered a reliable detector of fetal abnormality [4]. However, the sensitivity of karyotyping depends on the number of cells established in a particular culture, and results are usually not available for 3-4 days or more. Furthermore, it is very difficult to identify chromosome microdeletions. In addition to karyotype analysis, fluorescence *in situ *hybridization (FISH), an easy-to-handle, rapid, and highly sensitive tool for genetic analysis, has been developed in the past two decades [5-14]. Recently, AneuVysion FISH analysis has become the most common rapid screening method for prenatal or neonatal aneuploidies in a clinical setting. In most laboratories, AneuVysion analysis for prenatal testing costs between $300 and $350. However, FISH is labor-intensive and not easily applicable to a large number of samples in clinical diagnostic settings.

Multiplex quantitative fluorescence PCR (QF-PCR) provides the possibility to detect copy number variation of chromosomal sequences in several hours [12,15-19]. It also has the advantage of being much cheaper and allowing the simultaneous processing of larger numbers of samples than FISH and karyotyping analysis. However, the presence of multiple primer pairs in a PCR reaction reduces the reliability of the quantification. To solve these technical problems, multiplex ligation-dependent probe amplification (MLPA) has emerged as an alternative to standard PCR-based techniques for detection of the chromosome aneuploidies [20-22]. It allows for relative quantification of up to 50 different target sequences in one reaction and does not require living cells or cell culture. It is less labor-intensive and less expensive compared to karyotyping and FISH. Therefore, MLPA has been widely applied for molecular diagnosis of genetic diseases such as DMD, Spinocerebellar ataxia type 15 and chromosomal aneuploidies [21,23-26]. Moreover, a commercial MLPA kit based on length discrimination of the ligation products for detection of aneuploidies on chromosomes 13, 18, 21, X and Y has been developed. Its turn-around time can be as rapid as 30 hours.

A primary drawback of MLPA is its dependence on length-based discrimination of the ligation products. To differentiate between amplification products, the probes contain a non-hybridizing stuffer sequence of variable length. Therefore, MLPA limits the number of probes to 50 pairs or fewer. The size differences complicate the essential quantitative PCR step, since smaller fragments are amplified more efficiently. To resolve these technical hurdles, we have recently developed a universal flow-through array to quantify the MLPA amplification products, and successfully applied the array-MLPA assay for detection of deletions and duplications in Duchenne muscular dystrophy patients [27].

To determine whether the array-MLPA format can be used in clinical diagnostic settings to screen for common aneuploidies, we performed array-MLPA analysis on 161 peripheral blood and 12 amniotic fluid samples. Moreover, we confirmed the genotypes obtained on array-MLPA by G-banded karyotype analysis.

## Methods

### Patient materials

The peripheral blood samples were collected from 76 unrelated patients and 85 healthy individuals from Shanghai Children's Hospital. Twelve amniotic fluid samples (15-20 ml/sample) without blood contamination were obtained from the Institute of Medical Genetics in Henan Provincial People's Hospital. The amniotic fluid samples were collected from cases with abnormal maternal serum screening results. Informed consent was obtained from each participant. The study was approved by the ethics committee of Shanghai Children's Hospital.

Genomic DNA was extracted from peripheral blood or amniotic fluid cells (5 ml/sample) using the phenol/chloroform method. The concentration and quality of the DNA was estimated by measuring the optical density (OD) at 260 nm and 280 nm using a spectrophotometer (DU800, Beckman, US). Each sample was checked on gel electrophoresis. A DNA sample was discarded for further analyses if OD 260/280 ratio was not within the normal range (1.8-2.0). Additionally, the concentration of each DNA sample was standardized to 100 ng/μL. The data processing of the array-MLPA tests were performed without the knowledge of karyotype results.

### MLPA probe design and reaction

The design of the assay probes was essentially as described for array-MLPA [27]. The target sequences on chromosomes 13, 18, 21, X and Y were designed using the Ensembl genome browser (http://www.ensembl.org). Additionally, the target sequences of 8 autosomal genes on chromosomes 2, 4, 5, 11, 12, and 15 were designed as control probes. Ultimately, 116 pairs of MLPA probes covering chromosomes 13 (probe n = 28), 18 (probe n = 26), 21 (probe n = 22), X (probe n = 22), Y (probe n = 10) and 8 control probes were included in the study. The accession numbers and probe sequences are shown in Additional file 1, Table S1.

All of the oligonucleotides were chemically synthesized (Illumina, CA, USA) in a salt-free environment (25 nmol scale) and used without further purification. The probe mix was prepared at a final concentration of 4 nM per probe. MLPA reagents were purchased from MRC-Holland (Amsterdam, The Netherlands). The MLPA reaction was performed using a standard protocol described previously [27].

### Universal flow-through array and hybridization

In this study, a 4-well universal flow-through array with 124 pairs of 20-mer oligonucleotide tag-probes was used (PamGene, Den Bosch, The Netherlands). The preparation of the array using a porous aluminum-oxide substrate was performed as previously described [27-29]. In each of the arrays up to 400 probes can be spotted on the substrate. In comparison with two-dimensional geometry, the reactive surface in the porous substrate is increased approximately 500-fold. During hybridization, samples are actively pumped back and forth through the porous structure, resulting in a hybridization time of 5-30 minutes. Schematic depiction of the array technology is shown in Additional file 2, Figure S1.

The array hybridization was performed using a standard protocol described previously [27]. A single chip contained 4 identical arrays. One normal control and three other samples were analyzed in parallel. After washing the arrays, images were recorded at 500 ms, 1000 ms, 1500 ms and 3000 ms exposure time using a Cy5 filter set. Additionally, three independent experiments for each of 30 normal control individuals (15 males and 15 females) were performed to determine the accuracy of array-MLPA and the distributions of the normal copy numbers.

### Data analysis

Each array image was converted into spot intensity values using BioNavigator software (PamGene, Den Bosch, The Netherlands). Pixel-by-pixel cross-correlation was used to segment signal, background and artifact pixel populations. Median signal intensities were obtained for each spot on the array and local background was subtracted. A cutoff value for a positive signal was defined as three times above the standard deviation (SD) of the background. The mean signal of duplicate spots on each array was normalized using the mean signal of the eight control autosomal genes. Copy numbers of chromosomes 13, 18, 21, X and Y were computed as the ratio of test signal to autosomal control signal for each sample. The copy number data were exported into Microsoft Excel (Microsoft, Redmond, WA), where the median, SD and coefficients of variation (CV) of these chromosomes were calculated.

Normal copy number values for chromosomes 13, 18, 21 and X were defined between 0.85 and 1.15 in normal females, whereas the normal values of chromosomes X and Y were defined between 0.35 and 0.65 in normal males. Chromosomal trisomy was considered if the copy number was measured as 1.35 to 1.65 in a sample. The XXY type was detected if the copy number of chromosome X ranged from 0.85 to 1.15. When the ratio was below 0.65 in female patients, Turner's syndrome was identified. Samples that showed an average CV above 15% were considered to be unreliable.

### Karyotype analysis

Peripheral blood cells or amniotic fluid cells (10-15 ml/sample) were cultured according to the standard techniques on culture slides followed the method described by ISCN [28]. G banding was used for analysis and more than 20 metaphase chromosomes were routinely investigated. The results derived from array-MLPA and karyotyping were compared.

### Deletion confirmation

Y-chromosome-specific PCRs for *SRY*, *TSPY*, *RBMY *genes were performed to confirm deletions on the mosaic samples detected by array-MLPA. The primer sequences are listed in Table 1. The PCR reaction conditions were 95°C, 5 min followed by 33 cycles of 95°C, 1 min, 60°C, 1 min (55°C for TSPY gene) and 72°C, 2 min along with final extension at 72°C for 10 min. The PCR product was then subjected to 1% agarose gel for electrophoretic separation.

**Table 1:** PCR primers for deletion confirmation

Gene	Primer sequence	Annealing Temperature (°C)	PCR product length (bp)
SRY	5'-GAGACTCAGACAGCGAAGTA-3'	60	3155
	5'-ACGTCCAGGATAGAGTGAAG-3'		
TSPY	5'-TTACCTCCGTACCATCTACC-3'	55	3396
	5'-GAAGTCAGCCTCCAACTAAG-3'		
RBMY	5'-CCGTTATCCTCTTCAGTCAC-3'	60	3174
	5'-CATCTAGAGGCCATGCATAC-3'		

## Results

**Figure 1:**
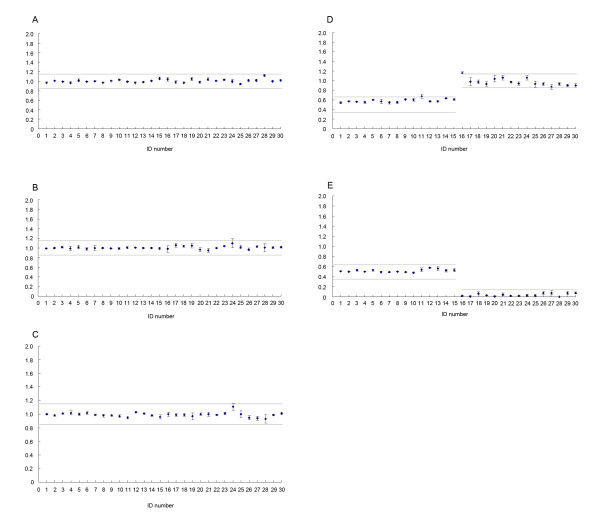
**Array-MLPA analysis of normal controls (15 normal females and 15 normal males)**. The average copy numbers on chromosomes 13, 18, 21, X and Y were shown in A, B, C, D and E, respectively. The normal distributions of copy number in the control individuals were shown with two grey lines. Error bars represented the corresponding standard deviation (SD) of the copy numbers for the MLPA probes covering each chromosome.

**Figure 2:**
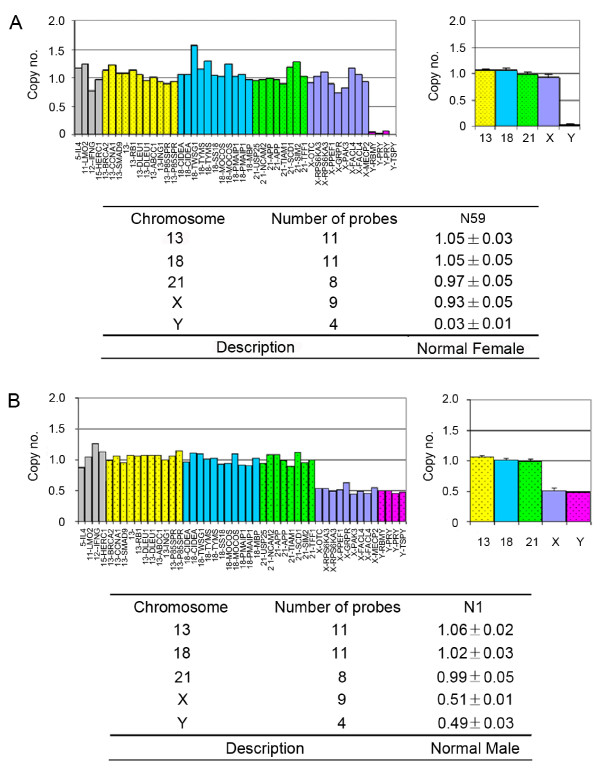
**A normal female and normal male were analyzed with array-MLPA**. The left figures showed the relative signal of each probe. The probes on chromosomes 13, 18, 21, X and Y were depicted in yellow, blue, green, purple and purplish red, respectively. Eight autosomal control probes were shown in grey. The right figures showed the average copy numbers on each chromosome. Error bars represented the corresponding SD of the copy numbers on the MLPA probes covering each chromosome. The copy number of each chromosome was listed in the lower tables. In the female, the copy numbers on chromosomes 13, 18, 21 and X were defined between 0.85 and 1.15. In the male, the copy numbers on chromosomes 13, 18 and 21 were nearly 1, while the copy numbers on chromosomes X and Y were approximately 0.5.

Array-MLPA is not a length-based discrimination method, which can overcome the main drawback of MLPA. The sensitivity and specificity of array-MLPA is based on the universal flow-through array hybridization and data analysis. To determine the reliability of array-MLPA, 30 normal control individuals (15 males and 15 females) were first tested with replicate measurements. The mean standard deviation across the 30 control individuals was 0.024, indicating the low inter-individual variability on the array-MLPA test. All probes designed on chromosomes 13, 18, 21 and X of female samples had an average relative signal of about 1.0, whereas the average signal of chromosomes X and Y in males was approximately 0.5. The copy numbers of almost all the controls were located within the normal distributions ( two grey lines in Figure 1). The intra-assay standard deviations of the probe signals on chromosome 13, 18, 21, X and Y ranged from 0.018 to 0.031. Examples of the copy numbers on array-MLPA for a normal female and a normal male are shown in Figure 2. The average gene copy numbers on the five chromosomes in the normal controls are listed in Additional file 3, Table S2. Overall, 89.4% of the relative probe signals in the normal controls were within the normal range (0.85 to 1.15).

**Figure 3:**
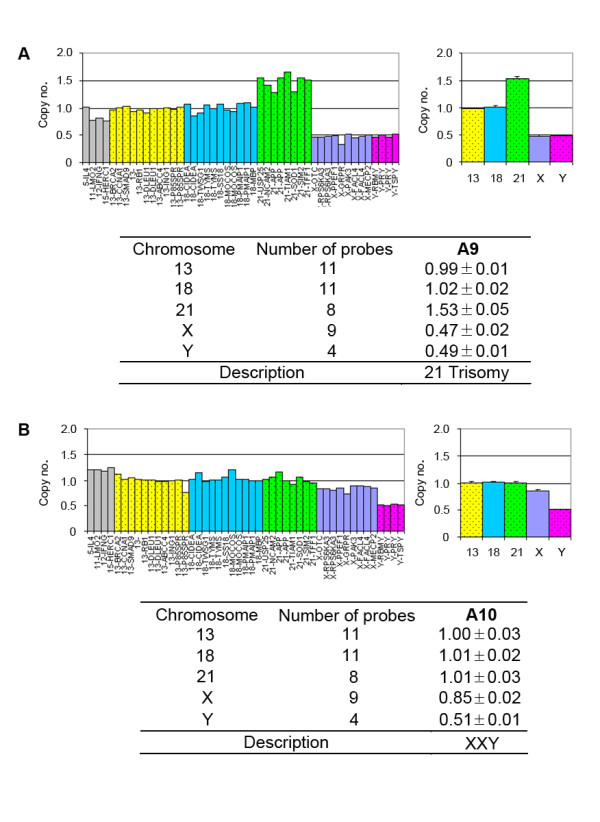
**The male fetus with trisomy 21 and XXY were analyzed with array-MLPA**. The left figures showed the relative signal of each probe. The probes on chromosomes 13, 18, 21, X and Y were depicted in yellow, blue, green, purple and purplish red, respectively. Eight autosomal control probes were shown in grey. The right figures showed the average copy numbers on each chromosome. Error bars represented the corresponding SD of the copy numbers on the MLPA probes covering each chromosome. The copy number of each chromosome was listed in the tables.

Next, array-MLPA was performed on 161 peripheral blood and 12 amniotic fluid samples including the 30 normal controls described above. Karyotype analysis was independently carried out on these samples. The results obtained by array-MLPA and the corresponding G-banded karyotypes were presented in Table 2. Of 173 tested samples, 169 (97.7%) including 12 amniotic fluid samples were correctly identified by array-MLPA. Figure 3 showed the abnormal copy numbers on chromosome 21 or X for two fetus samples. One was a male fetus (A9) in which the average copy number of chromosome 21 reached 1.53 indicating trisomy 21 (Figure 3A), while another was a male fetus (A10) in which the copy number on the X chromosome was 0.85 ±0.02 suggesting XXY (Figure 3B). However, four abnormal karyotypes including 46,XX,-14,+t(14;21);(p11.2;p11.2); 46,XY,rec(14)(18qter→18q22::14qter)pat; 46,XY,inv(9)(p12q21) and 46,XY,t(1;7)(p36.3;p13) could not be detected by array-MLPA (Table 2). A comparison of the results from array-MLPA and karyotyping was summarized in Table 3.

**Table 2:** Summary of the results of array-MLPA and karyotype analysis

Sample ID	Array-MLPA	Karyotype	Sample type
N1-N53	Normal 13, 18, 21, X, Y	46,XY	
N54-N85	Normal 13, 18, 21, X	46,XX	
B1-B4	Monosomy X	45,X	
B5-B6	Monosomy X+TSPY signals	45,X/45,X,+mar?	
B7	Monosomy X+RBMY, TSPY signals	45,X/46,XY	
B8-B9	Monosomy X+ RBMY, TSPY signals	45,X/46,XY(Yq-?)	
B10	Abnormal monosomy X (X_R _= 0.71)	45,X/46,X,i(X)(q10)	
B11	Abnormal monosomy X (X_R _= 0.72)	45,X/47,XXX	Blood
B12-B29	Trisomy 21 (21_R _= 1.36 to1.62)	47,XX,+21	
B30	Normal 13, 18, 21, X, Y	46,XY,rec(14)(18qter→18q22::14qter)pat	
B31-B72	Trisomy 21 (21_R _= 1.37 to1.56)	47,XY,+21	
B73	Male with extra X	47,XXY	
B74	Normal 13, 18, 21, X	46,XX,-14,+t(14;21);(p11.2;p11.2)	
B75	Normal 13, 18, 21, X, Y	46,XY,inv(9)(p12q21)	
B76	Normal 13, 18, 21, X, Y	46,XY,t(1;7)(p36.3;p13)	

A1-A4	Trisomy 21 (21_R _= 1.38 to1.52)	47,XX,+21	Amniotic fluid
A5-A9	Trisomy 21 (21_R _= 1.38 to1.53)	47,XY,+21	
A10-A11	Male with extra X (X_R _= 0.85, Y_R _= 0.51)	47,XXY	
A12	Normal 13, 18, 21, X, Y	46,XY	

**Table 3:** Comparison of the results from array-MLPA and karyotyping

	Blood	Amniotic fluid	Total	Mismatch genotype
Normal control	85	1	86	0/86 (0%)
Nonmosaic aneuploidy	69	11	80	4/80 (5%)
Mosaic aneuploidy	7	0	7	0/7 (0%)
Total	161	12	173	4/173 (2.3%)

**Figure 4:**
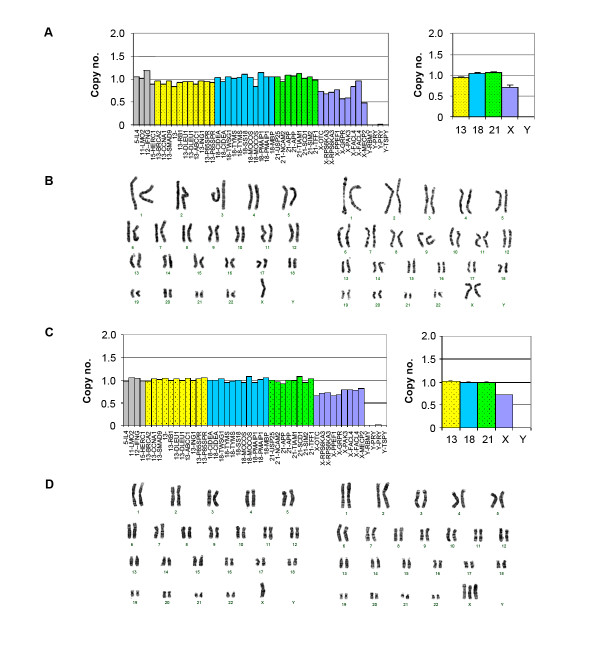
**Array-MLPA analysis of chromosome X monosomy mosaicism**. (A) A female patient (B10) with mosaicism. The average copy number on chromosome X was 0.71. (B) G-banding analysis revealed the patient had the karyotype 45,X/46,X,i(X)(q10). (C) A female patient (B11) with mosaicism. The average copy number on chromosome X was 0.72. (D) G-banding analysis revealed the patient had the karyotype 45,X(70%)/47,XXX (30%). Error bars represented the corresponding SD of the copy numbers on the MLPA probes covering each chromosome.

In this study, two cases (B10 and B11) with chromosome X monosomy mosaicism were identified successfully by array-MLPA (Table 2). For mosaic samples, the ratio reflects the extent of mosaicism, e.g. ratio 0.75 for a 50% chromosome X monosomy in a female sample. The results showed that the average copy numbers of chromosome X were 0.71 and 0.72 in the two samples. This suggested that approximately 50% of the sample cell population carried chromosome X monosomy. G-banded karyotype analysis showed that their chromosome karyotypes were 45,X/46,X,i(X)(q10) and 45,X/47,XXX for B10 and B11 respectively, which were basically consistent with array-MLPA results (Figure 4).

**Figure 5:**
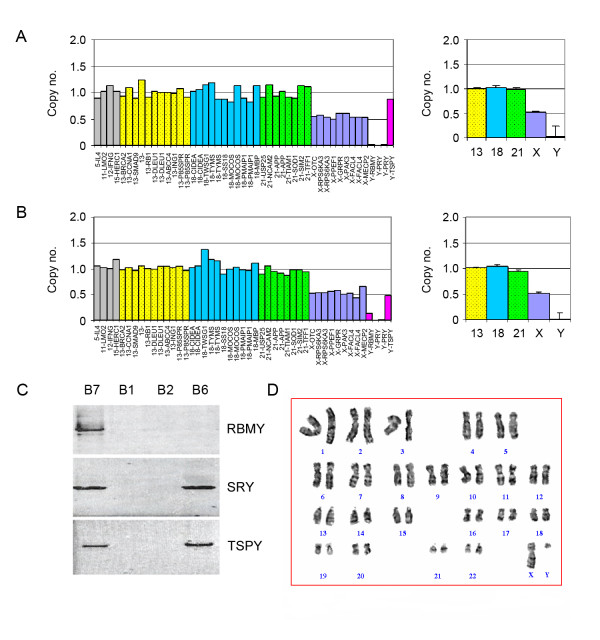
**Array-MLPA analysis of chromosome X monosomy patients with unknown marker chromosomes**. (A) Strong hybridization signals for *TSPY *were detected in sample B6. (B) Positive signals for *RBMY *and *TSPY *were found in sample B7. (C) PCR analysis of *RBMY, SRY *and *TSPY *detects the unknown marker chromosome detected with G-banding analysis. (D) The unknown marker chromosome in the sample B6 was derived from chromosome Y.

Additionally, five cases (B5-B9) with exceptive sex chromosome aneuploidies were identified using the criterion that at least four of ten Y chromosome-specific probes detected an abnormality. The probe signals in the *TSPY *gene were identified in all of the five cases (B5-B9). While the signals in the *RBMY *gene were detected in three samples (B7-B9). Subsequently, the G-banding analysis revealed that they were chromosome X monosomy mosaics with chromosome Y or unknown marker chromosome (mar) (Table 2). Their karyotypes were 45,X/45,X, mar for samples B5-B6, 45,X/46,XY for the sample B7 and 45,X/46,XY(Yq-) for samples B8-B9, respectively. PCR analysis of the *RBMY*, *SRY *and *TSPY *genes confirmed that the unknown marker chromosome found in samples B5 and B6 was indeed a part of chromosome Y (Figure 5).

## Discussion

Trisomy of chromosome 21, 13, or 18 as well as sex chromosome aneuploidy are the main genetic diseases of newborns. So far, there is no effective treatment for these diseases. Therefore, it is crucial to develop a rapid, reliable and highly sensitive molecular diagnostic technique. In this study, a 4-well universal flow-through array based on a three-dimensional aluminum-oxide substrate was used to analyze MLPA amplification products (array-MLPA). The main advantages of array-MLPA over other techniques such as karyotype analysis, FISH and multiplex PCR, are its simplicity, speed, and analysis of 124 detector probes in parallel. Four different samples for 124 genetic markers can be tested all at once within 6 hours starting from fresh blood or amniotic fluid samples. The cost of the universal chip is limited to $50 per sample. An additional advantage of this method is the automated measurement and data analysis. Compared to AneuVysion FISH analysis, the array-MLPA system demonstrates at least 4-fold shorter assay time and at least 6-fold less assay cost. The reproducibility and accuracy of the array technology were demonstrated previously in studies on gene expression profiling [29,30] and quantification of PMP22 gene copy number [31] and detection of DMD genomic rearrangements [27].

To further investigate the applicability of array-MLPA for rapid chromosomal aneuploidy screening, especially in fetuses at risk for Down's syndrome, we performed array-MLPA analysis on 161 peripheral blood and 12 amniotic fluid samples. Our results showed that 169 of 173 (97.7%) samples including all the amniotic fluid samples identified by array-MLPA were concordant with karyotype analysis. Our study demonstrates the successful application and strong potential of array-MLPA in clinical diagnosis and prenatal testing for rapid chromosomal aneuploidy screening. Further validation studies must be performed to ensure the clinical applicability of our array-MLPA assay.

To improve the reliability of detection, we designed two different probes per targeted gene, located in the 5' and 3' region of the same gene, and the average values of two probes were used to calculate the copy number of the gene. The data indicated that 89.4% of the relative probe signals from array-MLPA ranged from 0.85 to 1.15 in the normal controls. In the previous MLPA analysis [26], the normal region was usually set defined as 0.7 to 1.3. This indicates that array-MLPA is more accurate than the reported results with conventional MLPA for the determination of copy number.

It is well-known that intrinsic sensitivity limitations of MLPA make it unsuitable for reliable detection of mosaicism. It has become possible to detect the mosaicism of chromosomal aneuploidies by array-MLPA due to its higher accuracy. In this study, we identified sex chromosome mosaicism in two patients by array-MLPA. In one case (B10), the average copy number on chromosome X was 0.71 revealing approximately 50% mosaic rate of chromosome X. The result was concordant with that of conventional cytogenetic analysis. In another case (B11), a similar result was observed on array-MLPA. However, G-banded karyotype analysis indicated the chromosome karyotypes 45,X(70%)/47,XXX(30%). The result showed that there were polyploid cells in the patient B11. This suggests array-MLPA might have a bias in detection. Array-MLPA appeared to be more suitable for detecting mosaicism of monosomy than polyploidy.

In addition to X chromosome aneuploidies, unknown marker chromosomes in four cases (B5, B6, B8 and B9) were found by karyotype analysis, but G-banding did not distinguish their origins. Finally, we identified the regions of these unknown marker chromosomes by array-MLPA. Two of the four cases were confirmed by Y-chromosome-specific PCR. In another case (B7), abnormality on the Y chromosome was identified by both array-MLPA and G-banding analysis. In this study, we designed ten pairs of probes to cover the long and short arms of chromosome Y. The data obtained on array-MLPA and PCR analysis indicate the presence of Y chromosome-specific probe signals, especially *TSPY *probes. Our study demonstrates the power of array-MLPA for detecting small abnormalities on chromosomes.

However, four samples with chromosome translocations or inversions were not identified by array-MLPA since the recombination regions were not covered by the MLPA probes. Currently, we have only used one (Cy5) of the four available detection wavelengths for screening chromosomes 13, 18, 21, X, and Y. Using three additional sets of amplification primers, each labelled with a different fluorophore [32], it should be simple to expand the number of MLPA probes for other chromosomal abnormalities with potential clinical significance. Still, array-MLPA cannot completely replace conventional cytogenetic analysis. It is suitable to be used as a screening test in conjunction with karyotype analysis for diagnosis.

## Conclusions

Our study demonstrates the successful application of array-MLPA for clinical molecular diagnosis with rapid and sensitive screening for chromosomal aneuploidies. Furthermore, we have developed a simple and rapid procedure for screening copy numbers on chromosomes 13, 18, 21, X, and Y using array-MLPA. Since array processing and data analysis are fully automated, array-MLPA should be suitable for large scale testing for chromosome aneuploidies in clinical diagnostic settings.

## Abbreviations

MLPA: multiplex ligation-dependent probe amplification; FISH: fluorescence *in situ *hybridization; QF-PCR: Multiplex quantitative fluorescence PCR; CV: Coefficient of variation; ISCN: An international system for human cytogenetic nomenclature

## Competing interests

The authors declare that they have no competing interests.

## Authors' contributions

JY, ZR, Y-TW and SL were involved in patient and control subject recruitments. MM, RvB and YW participated in the design of MLPA probes and analysis software. JY, MX, CX, and DZ were involved in the array-MLPA experiments and data analysis. YH participated in the karyotyping analysis. FZ, YW and YZ conceived of the study, and participated in its design and coordination. JY, ZR, FZ and YW wrote the manuscript. All authors contributed to and have approved the final manuscript.

## Pre-publication history

The pre-publication history for this paper can be accessed here:

http://www.biomedcentral.com/1471-2350/12/68/prepub

## Supplementary Material

Additional file 1 Table S1: Probe sequence for chromosomal aneuploidy Click here for file

Additional file 2 **Figure S1: Schematic depiction of the array technology**. (A) Four-well array system was used in the study. Each array contained 412 spots, and the oligonucleotide was immobilized on an aluminum-oxide substrate. The substrate had a thickness of 60 μm with long capillary pores. The diameter of an individual pore was between 100 and 200 nm. A single spot occupied about 100,000 pores of the substrate. (B) Raw image obtained on an array. These images were recorded at 500 ms, 1000 ms, 1500 ms and 3000 ms exposure time using a Cy5 filter set. This array design contained 120 probes spotted in duplicate (240 spots), including 116 universal tag-probe oligonucleotides for different chromosomes, two exogenous target ArrayControl RNA Spikes (AM1780, Ambion, Austin, TX) oligonucleotides used as a negative control for array hybridization, and two Cy5-reference oligonucleotides. Click here for file

Additional file 3 Table S2: Gene copy numbers on array-MLPA in normal controls Click here for file
