# Supplementary material for: Rapid screening for chromosomal aneuploidies using array-MLPA
Source: BMC Med Genet. 2011 May 17;12:68. doi: 10.1186/1471-2350-12-68 (PMC3111339; doi:10.1186/1471-2350-12-68)
Supplement: Additional file 1 — Table S1: Probe sequence for chromosomal aneuploidy [file 1471-2350-12-68-S1.DOC]

**Table S1. Probe sequence for chromosomal aneuploidy**

| **Chromosome location** | **Gene** | **Accession number** | **Lest probe sequence** | **Right probe sequence** |
| --- | --- | --- | --- | --- |
| 2q13 | IL1A | NM_000575 | *CCAGTGAATTGTAATACGAC*GTAGGAGTGGGTAGAAGGTGGATGCCTGAGATACCCAAAACCATC | ACAGGTAGTGAGACCAACCTCCTCT*CTTTTGTTCCCTTTAGTGAG* |
| 4q26-q27 | IL2 | NM_000586 | *CCAGTGAATTGTAATACGAC*GTGGTGATGAAGGTGAGAGGCCTCAACTCCTGCCACAATGTACAG | GATGCAACTCCTGTCTTGCATTGC*CTTTTGTTCCCTTTAGTGAG* |
| 5q23-q31 | IL4 | NM_172348 | *CCAGTGAATTGTAATACGAC*GTGGGTGAGGAGGTTGTAAGCTGCAAATCGACACCTATTAATGGG | TCTCACCTCCCAACTGCTTCCC*CTTTTGTTCCCTTTAGTGAG* |
| 11p13 | LMO2 | NM_005574 | *CCAGTGAATTGTAATACGAC*CACCGCTACTTCGCTACATCCCTGTGACAAGCGGATTCGTGCCTA | TGAGATGACAATGCGGGTGAAAGAC*CTTTTGTTCCCTTTAGTGAG* |
| 12q24.1 | IFNG | NM_000619 | *CCAGTGAATTGTAATACGAC*CACACCACCCAACCCTACTTCTCATCCAAGTGATGGCTGAACTGT | CGCCAGCAGCTAAAACAGGGAAGCG*CTTTTGTTCCCTTTAGTGAG* |
| 12p12.1 | LRMP | NM_006152 | *CCAGTGAATTGTAATACGAC*CAGAGGAGCAGAAACGGTACGGACTCATGGACGTCTCTAGAACAT | ATCTTGTGGCCATTTACCAGACTCC*CTTTTGTTCCCTTTAGTGAG* |
| 12p13 | CD27 | NM_001242 | *CCAGTGAATTGTAATACGAC*CGAGCAAGAGGGATGAACACAAAGTCCTGTGGAGCCTGCAGAGCC | TTGTCGTTACAGCTGCCCCAGGGAG*CTTTTGTTCCCTTTAGTGAG* |
| 15q22 | HERC1 | NM_003922 | *CCAGTGAATTGTAATACGAC*CACCTCCCAAACCTCAACCAAGGTGAAGCAGCAGCAGCATTCTCC | GGAGAGCAGTTCTGGTTCAGCAGATGT*CTTTTGTTCCCTTTAGTGAG* |
| 13q12.3 | BRCA2 | NM_000059 | *CCAGTGAATTGTAATACGAC*GCAGACAAGGAAGAACAGCCATTCAAAGAGCAAGGGCTGACTCTG | CCGCTGTACCAATCTCCTGTAAAAG*CTTTTGTTCCCTTTAGTGAG* |
| 13q12.3 | BRCA2 | NM_000059 | *CCAGTGAATTGTAATACGAC*CAACCTCTACTCCTTCACCCTCTGTGTGACACTCCAGGTGTGGAT | CCAAAGCTTATTTCTAGAATTTGGG*CTTTTGTTCCCTTTAGTGAG* |
| 13q12.3-q13 | CCNA1 | NM_003914 | *CCAGTGAATTGTAATACGAC*CCTCATCCACATACGCAACCGTCACTTGGGATGGAGACCGGCTTT | CCCGCAATCATGTACCCTGGATCTT*CTTTTGTTCCCTTTAGTGAG* |
| 13q12.3-q13 | CCNA1 | NM_003914 | *CCAGTGAATTGTAATACGAC*CAACCACGCTCTCTTACTACCACTTCCAGAACTTCACCTCCATAT | CAGAAGTGCCAATAATCGTCATAGG*CTTTTGTTCCCTTTAGTGAG* |
| 13q12-q14 | SMAD9 | NM_005905 | *CCAGTGAATTGTAATACGAC*CAAGCCAAGAACTCCGTCACAGTGAAGAGACTGCTAGGCTGGAAG | CAAGGAGATGAAGAGGAAAAGTGGG*CTTTTGTTCCCTTTAGTGAG* |
| 13q12-q14 | SMAD9 | NM_005905 | *CCAGTGAATTGTAATACGAC*CAATGTCACGACCCAGTCACGACTCAGATGGGCTCTCCACATAAC | CCCATTTCTTCAGTGTCTTAACAGTC*CTTTTGTTCCCTTTAGTGAG* |
| 13q13 | NBEA | NM_015678 | *CCAGTGAATTGTAATACGAC*GACGACCAAGACGAAACGACGGATGATTAACCCTTCGGTGCCGAT | CCGCAACATCCGGATGAAATTCGCA*CTTTTGTTCCCTTTAGTGAG* |
| 13q13 | NBEA | NM_015678 | *CCAGTGAATTGTAATACGAC*GAAGGCACGACAGAAGAGACGGCATTATGAGCATCAGAACAGATACT | GAAGATAAAGGAAGAACCAAAAGCC*CTTTTGTTCCCTTTAGTGAG* |
| 13q13 | DCAMKL1 | NM_004734 | *CCAGTGAATTGTAATACGAC*CCAGTCCACCACCTAGTCTAAGCTCCGAGAAGAAGGCCAAGAAAG | TTCGTTTCTATCGAAACGGAGATCG*CTTTTGTTCCCTTTAGTGAG* |
| 13q13 | DCAMKL1 | NM_004734 | *CCAGTGAATTGTAATACGAC*GAGAACGCACAGTGACGTGAACCGCTCTTGATAAGGAGAGGCAGG | TTTTCCGACGAAGACGCAACCAGGA*CTTTTGTTCCCTTTAGTGAG* |
| 13q14.2 | RB1 | NM_000321 | *CCAGTGAATTGTAATACGAC*CTACCCATCACGATCTCCACAATGGTTCACCTCGAACACCCAGGC | GAGGTCAGAACAGGAGTGCACGGAT*CTTTTGTTCCCTTTAGTGAG* |
| 13q14.2 | RB1 | NM_000321 | *CCAGTGAATTGTAATACGAC*GGAATGCACGAACACCACACATCAGATGGTATGTAACAGCGACCG | TGTGCTCAAAAGAAGTGCTGAAGGA*CTTTTGTTCCCTTTAGTGAG* |
| 13q14.3 | DLEU1 | NR_002605 | *CCAGTGAATTGTAATACGAC*GGGTGACCACGAGTTGATGAGCCCACAGGCATTTAGTCTACGTTG | GAGGTAAACAAATACGGGTCCTGCT*CTTTTGTTCCCTTTAGTGAG* |
| 13q14.3 | DLEU1 | NR_002605 | *CCAGTGAATTGTAATACGAC*GAGGAGGTGATGGTGATGGAGTTCCTATCAAAGTGTCGTATCTGG | GAGATGACCTGCCTTATCCTGTTCT*CTTTTGTTCCCTTTAGTGAG* |
| 13q22 | DACH | AF356492 | *CCAGTGAATTGTAATACGAC*GGGTGTAGGTTGGGATGAGATCCCCCTTCTCTCCCTTTCTCCTTC | TCGTTCTCTCCCGGAGTTGTTGTTG*CTTTTGTTCCCTTTAGTGAG* |
| 13q22 | DACH | AF356492 | *CCAGTGAATTGTAATACGAC*CTCCCAGGCACAAGTACAGATCTCCATTGATTTTCACCTGTGGTT | CATACCAGAGACCTGAGAATGTTTG*CTTTTGTTCCCTTTAGTGAG* |
| 13q22.1 | KIAA1008 | NM_014953 | *CCAGTGAATTGTAATACGAC*GAGCCAGCAAGACACAGAACTCTGCCCGCAACCGCACTACTTGCT | GCCCGACACTAATGTGTTACTGCAC*CTTTTGTTCCCTTTAGTGAG* |
| 13q22.1 | KIAA1008 | NM_014953 | *CCAGTGAATTGTAATACGAC*CAACACCGAGTAGCTGCAACTCACTTAAATACAGTGACAGGACAG | CAACTTCAGGGATCTGTAAAGATCA*CTTTTGTTCCCTTTAGTGAG* |
| 13q31 | ABCC4 | AY207008 | *CCAGTGAATTGTAATACGAC*GCATCACGGTGAGCTGAAGAATCAATGTACTTGCCAGGGAGAGCC | CAAGTCCTTCAAACCTCCTCCTTTT*CTTTTGTTCCCTTTAGTGAG* |
| 13q31 | ABCC4 | AY207008 | *CCAGTGAATTGTAATACGAC*CGGAGCTTCAAAGCACGTCATTAAAATGTGCTGTGCTTCCCACCC | TTGTCAGAGGGAAGGGTGGCTATGT*CTTTTGTTCCCTTTAGTGAG* |
| 13q31.1 | SPRY2 | NM_005842 | *CCAGTGAATTGTAATACGAC*CACCTCGTTCATACACCACCTAAGCCTGCTGGAGTGACCACACTT | CCAAGACCTGATGGAGGCCAGAGCT*CTTTTGTTCCCTTTAGTGAG* |
| 13q31.1 | SPRY2 | NM_005842 | *CCAGTGAATTGTAATACGAC*CCCTCCTCTTCCAACTAACCTCCAGCCAAGGGTTGCCTTAAATTG | TGCCAGGGGTGTTATGACCGGGTTA*CTTTTGTTCCCTTTAGTGAG* |
| 13q32-q33 | TPP2 | NM_003291 | *CCAGTGAATTGTAATACGAC*CAAGCGAACGGACGAGAAACTGCGACTGAGGAGCCCTTCCCTT | TTCACGGTCTCCTGCCGAAGAAGGA*CTTTTGTTCCCTTTAGTGAG* |
| 13q32-q33 | TPP2 | NM_003291 | *CCAGTGAATTGTAATACGAC*CGCCTACGAGGAGACTTCTAGATGGACCCATTGTGCATCTTTTAC | TGAAAACTGGCTCCCCATCATGTAT*CTTTTGTTCCCTTTAGTGAG* |
| 13q34 | ING1 | NM_198219 | *CCAGTGAATTGTAATACGAC*CACTCCACTTGACCCTCTCAAGCAGCTCCACCTGGTGAACTATGT | GGAGGACTACCTGGACTCCATCGAG*CTTTTGTTCCCTTTAGTGAG* |
| 13q34 | ING1 | NM_198219 | *CCAGTGAATTGTAATACGAC*CCTCGATCTACCATCCCTCACCCAACGAACCCACGTACTGTCTGT | GCAACCAGGTCTCCTATGGGGAGAT*CTTTTGTTCCCTTTAGTGAG* |
| 13q34 | P85SPR | NM_003899 | *CCAGTGAATTGTAATACGAC*CCCTAACTCCCTCACCATCATTTGCACACTCGGACTTCAAAACTG | TTCCAGGGCCAGTATCGGAGTTTGG*CTTTTGTTCCCTTTAGTGAG* |
| 13q34 | P85SPR | NM_003899 | *CCAGTGAATTGTAATACGAC*CAGCATCAGCATCAGGTGCAGTCCTCAGTTCTTTCTGTTGAAGAC | CAGTTCTGAGGTGAAGCTGGGCACC*CTTTTGTTCCCTTTAGTGAG* |
| 18p11.2 | MC2R | NM_000529 | *CCAGTGAATTGTAATACGAC*CGACCAAGACGAGAACGAAGAAGTCAAGTCCAAGTAACATCCCCG | CCTTAACCACAAGCAGGAGAAATGA*CTTTTGTTCCCTTTAGTGAG* |
| 18p11.2 | MC2R | NM_000529 | *CCAGTGAATTGTAATACGAC*CACGGACCAAAGAGGAGAAGGGACGCATTCAAAAAGATGATCTTC | TGCAGCAGGTACTGGTAGAATGGCT*CTTTTGTTCCCTTTAGTGAG* |
| 18p11.21 | CIDEA | NM_001279 | *CCAGTGAATTGTAATACGAC*CGTGCGTTGGTCAAAGTTCCATCAGGCCCCTGACATTTATGGGAT | CACAGACTAAGCGAGTCCTGTTCAC*CTTTTGTTCCCTTTAGTGAG* |
| 18p11.21 | CIDEA | NM_001279 | *CCAGTGAATTGTAATACGAC*CTCTCCCATCAACACCATCCGATGACAAGGAAGAGCGGCCATCCC | TCCGGTCACAAGCCAAGGGCAGGTT*CTTTTGTTCCCTTTAGTGAG* |
| 18p11.3 | TWSG1 | NM_020648 | *CCAGTGAATTGTAATACGAC*CACATCTCCAGCGAACATGCGTTGCTGTGCTTACTCTAGCCATCC | TGATGTTCCTGACATGGCTTCCAGA*CTTTTGTTCCCTTTAGTGAG* |
| 18p11.3 | TWSG1 | NM_020648 | *CCAGTGAATTGTAATACGAC*CCATGCAGAACGTACTACGCTCGCTGGTTTCATAATGCCTGCTGC | GAGTGCATTGGTCCAGAATGTATTG*CTTTTGTTCCCTTTAGTGAG* |
| 18p11.32 | TYMS | NM_001071 | *CCAGTGAATTGTAATACGAC*GGTGTGAAGGGTAGGTAGAGGGAGCTGCAGTACCTGGGGCAGATC | CAACACATCCTCCGCTGCGGCGTCA*CTTTTGTTCCCTTTAGTGAG* |
| 18p11.32 | TYMS | NM_001071 | *CCAGTGAATTGTAATACGAC*GACTGTGGAAGCATCGTCAGCTTTGAGTTAACTCACTGAGGGTAT | CTGACAATGCTGAGGTTATGAACAA*CTTTTGTTCCCTTTAGTGAG* |
| 18q11.2 | SS18 | NM_001007559 | *CCAGTGAATTGTAATACGAC*GAACCGAGTGCTGCACAAAGAGCGCTCGGGACTTGCCGATAGTGG | TGACGGCGGCAACATGTCTGTGGCT*CTTTTGTTCCCTTTAGTGAG* |
| 18q11.2 | SS18 | NM_001007559 | *CCAGTGAATTGTAATACGAC*CTCCGCTGTGATCTCGTTTCTCCAGTAGCCAGTATCTATTAGCAG | CCATATTGTCACCTCAGCACTGTGG*CTTTTGTTCCCTTTAGTGAG* |
| 18q12 | MOCOS | NM_017947 | *CCAGTGAATTGTAATACGAC*CTTCTCAGGTCCGTTTGCTCGCACTTCCACACCACCGCAGAAGAC | TACACTGTGATCTTCACTGCCGGGA*CTTTTGTTCCCTTTAGTGAG* |
| 18q12 | MOCOS | NM_017947 | *CCAGTGAATTGTAATACGAC*CACCTCACTCCAACCTACTCGTCTGTAGGATCTCAGGTGCTCCCT | GTGTTGAAAGAGAATGTGGAAGGTC*CTTTTGTTCCCTTTAGTGAG* |
| 18q21.1 | MADH4 | NM_005359 | *CCAGTGAATTGTAATACGAC*GGACAGCAGGAAACAAGACGCGAATACACCAACAAGTAATGATGC | CTGTCTGAGCATTGTGCATAGTTTG*CTTTTGTTCCCTTTAGTGAG* |
| 18q21.1 | MADH4 | NM_005359 | *CCAGTGAATTGTAATACGAC*CAACGGAACCCAAAAGGGAGAGACTGAGGTCTTTTACCGTTGGGG | CCCTTAACCTTATCAGGATGGTGGA*CTTTTGTTCCCTTTAGTGAG* |
| 18q21.1-q21.32 | GRP | NM_002091 | *CCAGTGAATTGTAATACGAC*CAAAGACACCAACGACGAGGTCCTCCGAGGTCCGGGTCACCAGT | CTCTGCTCTTCCCAGCCTCTCCGG*CTTTTGTTCCCTTTAGTGAG* |
| 18q21.1-q21.32 | GRP | NM_002091 | *CCAGTGAATTGTAATACGAC*GGGAAGGTTAGAGGTGAAGGAACGTGAAGGAAGGAACCCCCAGCT | GAACCAGCAATGATAATGATGGCCT*CTTTTGTTCCCTTTAGTGAG* |
| 18q21.1-q21.32 | GRP | NM_002091 | *CCAGTGAATTGTAATACGAC*CTCAACCTCACCTCCAACTCCTTAATGGGGAAAAAGAGCACAGGG | GAGTCTTCTTCTGTTTCTGAGAGAG*CTTTTGTTCCCTTTAGTGAG* |
| 18q21.1-q21.32 | GRP | NM_002091 | *CCAGTGAATTGTAATACGAC*CCATCCTCACAACCTCCATCGGTTCTCAACGTGAAGGAAGGAACC | CCCAGCTGAACCAGCAATGATAATG*CTTTTGTTCCCTTTAGTGAG* |
| 18q21.3 | SERPINB2 | NM_002575 | *CCAGTGAATTGTAATACGAC*CACGCAGAAGAGTACGACAGTGAAACAATGGAGGATCTTTGTGTG | GCAAACACACTCTTTGCCCTCAATT*CTTTTGTTCCCTTTAGTGAG* |
| 18q21.3 | SERPINB2 | NM_002575 | *CCAGTGAATTGTAATACGAC*GAAAGCTACGACCAGCACAGGCGTGCTGCTTCTGCAAAAGATTTT | TGTAGATGAGCTGTGTGCCTCAGAA*CTTTTGTTCCCTTTAGTGAG* |
| 18q21.32 | PMAIP1 | NM_021127 | *CCAGTGAATTGTAATACGAC*GTCTGTTGCTCGTGGTTGTCGACTGTTCGTGTTCAGCTCGCGTCC | TGCAGCTGTCCGAGGTGCTCCAGTT*CTTTTGTTCCCTTTAGTGAG* |
| 18q21.32 | PMAIP1 | NM_021127 | *CCAGTGAATTGTAATACGAC*GTCCTCTGTTGTTTGCGGTCGCATCAAAAACTTGCATGAGGGGAC | TCCTTCAAAAGAGTTTTCTCAGGAGG*CTTTTGTTCCCTTTAGTGAG* |
| 18q23 | MBP | NM_001025101 | *CCAGTGAATTGTAATACGAC*CAACAACAGACAGAGCACGGAATCCATTCAGGATGGGAAACCACG | CAGGCAAACGAGAATTAAATGCCGA*CTTTTGTTCCCTTTAGTGAG* |
| 18q23 | MBP | NM_001025101 | *CCAGTGAATTGTAATACGAC*GGGTTGGAGTATGATCGAGGTAGACGCTGAAAACCCACCTGGTTC | CGGAATCCTGTCCTCAGCTTCTTAA*CTTTTGTTCCCTTTAGTGAG* |
| 18q23 | NFATC1 | NM_172390 | *CCAGTGAATTGTAATACGAC*CACGGAAGACAAGTCAGCAGTGCCAAGCACCAGCTTTCCAGTCCC | TTCCAAGTTTCCACTTGGCCCTGCG*CTTTTGTTCCCTTTAGTGAG* |
| 18q23 | NFATC1 | NM_172390 | *CCAGTGAATTGTAATACGAC*GAAGGAGAGCAAGGAACCAGCAGCGTGGCGTGTTGCACATTTAAC | TGTGTGATGTCCCGTTAGTGAGACC*CTTTTGTTCCCTTTAGTGAG* |
| 21q11 | STCH | NM_006948 | *CCAGTGAATTGTAATACGAC*CTGCTGGTCGTTCTGAACCTAGAGAGATGACGATCTTAGGATCGG | CTGTTTTGACTCTCCTGTTGGCCGG*CTTTTGTTCCCTTTAGTGAG* |
| 21q11 | STCH | NM_006948 | *CCAGTGAATTGTAATACGAC*GGGAAGGATGGTGGACATTGCAAGCAGGGATTGATGGAGGCTCTT | GGCCTCTCCAAGTCAGTGCTTTAGA*CTTTTGTTCCCTTTAGTGAG* |
| 21q11.2 | USP25 | NM_013396 | *CCAGTGAATTGTAATACGAC*GTCTGTCGTCGTCTTGTCTGTGAATCAACTGAGAGAAATTACGGG | GATTAATGACACCCAGATACTACAG*CTTTTGTTCCCTTTAGTGAG* |
| 21q11.2 | USP25 | NM_013396 | *CCAGTGAATTGTAATACGAC*GTTGCCTTGTCGAATCCCTGGTCCCTCAGTCGAACTCCTGCTGAT | GGAAGATAAACTGCACACTTTCCCT*CTTTTGTTCCCTTTAGTGAG* |
| 21q21.1 | NCAM2 | NM_004540 | *CCAGTGAATTGTAATACGAC*CCTCTGAGAACCTCTGGTCTGAACATGAGCCTCCTCCTCTCCTTC | TACCTGCTGGGGTTGCTTGTCAGTA*CTTTTGTTCCCTTTAGTGAG* |
| 21q21.1 | NCAM2 | NM_004540 | *CCAGTGAATTGTAATACGAC*CTCACCCTCTACCAAACCCTGTCAAGTCACACTCGTATGTGATGC | GGAAGGGGAGCCTATTCCAGAAATC*CTTTTGTTCCCTTTAGTGAG* |
| 21q21.3 | APP | NM_201414 | *CCAGTGAATTGTAATACGAC*GTGGAGTGTGAGTGGAGATGAGGTACCCACTGATGGTAATGC | TGGCCTGCTGGCTGAACCCCAGATT*CTTTTGTTCCCTTTAGTGAG* |
| 21q21.3 | APP | NM_201414 | *CCAGTGAATTGTAATACGAC*GGGTGGATCTGGATGGAATGCACAGCAGCCTCTGAAGTTGGACAG | CAAAACCATTGCTTCACTACCCATC*CTTTTGTTCCCTTTAGTGAG* |
| 21q22.11 | TIAM1 | NM_003253 | *CCAGTGAATTGTAATACGAC*GAGTTGAGAGGGAGGTAGTGCATGGGAAACGCAGAAAGTCAACAT | GTAGAGCACGAGTTTTATGGAGAAA*CTTTTGTTCCCTTTAGTGAG* |
| 21q22.11 | TIAM1 | NM_003253 | *CCAGTGAATTGTAATACGAC*GTCCGTCCTTCTTCTGTTGGAAGACTTTGCCCCCTCCAGGAAACT | GAACACTGAGATCTGACTGCGTCAC*CTTTTGTTCCCTTTAGTGAG* |
| 21q22.11 | SOD1 | NM_000454 | *CCAGTGAATTGTAATACGAC*GTTTCTGCCTGGTTGTGTGGCTCGGCGTGGCCTAGCGAGTTATGG | CGACGAAGGCCGTGTGCGTGCTGAA*CTTTTGTTCCCTTTAGTGAG* |
| 21q22.11 | SOD1 | NM_000454 | *CCAGTGAATTGTAATACGAC*GTGAAGAGGTGGGTGAATGGGTCGTTTGGCTTGTGGTGTAATTGG | GATCGCCCAATAAACATTCCCTTGG*CTTTTGTTCCCTTTAGTGAG* |
| 21q22.13 | SIM2 | NM_005069 | *CCAGTGAATTGTAATACGAC*GTAGGCTAGTGTTGGAAGGGAGAAGTCCAAGAATGCGGCCAAGAC | CAGGAGGGAGAAGGAAAATGGCGAG*CTTTTGTTCCCTTTAGTGAG* |
| 21q22.13 | SIM2 | NM_005069 | *CCAGTGAATTGTAATACGAC*CGCTTACGGTACGATCAAGGTGCGCACGACCTACATTAATTTATG | CAGAGACAGCTGTTTGAATTGGACC*CTTTTGTTCCCTTTAGTGAG* |
| 21q22.2 | DSCR8 | NM_203429 | *CCAGTGAATTGTAATACGAC*CTCTCGCTCTTCGGTTCGTTTACATTTTCCCAGACAGGAATTTGG | CTGCCAACAGGGAATTCTAAACAAC*CTTTTGTTCCCTTTAGTGAG* |
| 21q22.2 | DSCR8 | NM_203429 | *CCAGTGAATTGTAATACGAC*CGCTTGGTCCTTCTGTGCTTGCATTTGTTAAGCACCTACTGTGAG | TAGATGATCTCCTGTCAAAGACAGT*CTTTTGTTCCCTTTAGTGAG* |
| 21q22.2 | PCP4 | NM_006198 | *CCAGTGAATTGTAATACGAC*GGTGTTCTCCTGTTGCTGTGGCGACAAGGTGCTGGGGCAACCAAT | GGAAAAGACAAGACATCTGGTGAAA*CTTTTGTTCCCTTTAGTGAG* |
| 21q22.2 | PCP4 | NM_006198 | *CCAGTGAATTGTAATACGAC*GGATGAGAGTGGTAGGAGTGTCAGAAAATTCCAGAAGAAGAAGGC | TGGGTCTCAGTCCTAGTGGGAGAA*CTTTTGTTCCCTTTAGTGAG* |
| 21q22.3 | KIAA0179 | NM_015056 | *CCAGTGAATTGTAATACGAC*CTCCTGTTCGGTGGTCTTGTTGAAGAAGCTGCGCCAGTACATCAG | CGTGAAGACGCAGAGGGAGACAG*CTTTTGTTCCCTTTAGTGAG* |
| 21q22.3 | KIAA0179 | NM_015056 | *CCAGTGAATTGTAATACGAC*GTAAGACTCGACACGCTCGTTTTGAGCAGCGATTGCTGGATCATT | GATCTGTTTGAGGAAGTGTCTGACC*CTTTTGTTCCCTTTAGTGAG* |
| 21q22.3 | TFF1 | NM_003225 | *CCAGTGAATTGTAATACGAC*GGTAGGTGTGTGAGATAGGGGCAGAGAGGAGGCAATGGCCACCAT | GGAGAACAAGGTGATCTGCGCCCTG*CTTTTGTTCCCTTTAGTGAG* |
| 21q22.3 | TFF1 | NM_003225 | *CCAGTGAATTGTAATACGAC*GATGAGGTTGGATGTGAGGGGAGGAGTGTGAATTTTAGACACTTC | TGCAGGGATCTGCCTGCATCCTGAC*CTTTTGTTCCCTTTAGTGAG* |
| Xp11.4 | TM4SF2 | NM_004615 | *CCAGTGAATTGTAATACGAC*CCATCCTAGAGCTGCTATCCCATCGAGGAGAATGGAGACCAAACC | TGTGATAACCTGTCTCAAAACCCTC*CTTTTGTTCCCTTTAGTGAG* |
| Xp11.4 | TM4SF2 | NM_004615 | *CCAGTGAATTGTAATACGAC*CCACATCACTCACCTGTACCTTAATTGGCATGCTGCTGGCCTGCT | GTCTGTCCCGGTTCATCACGGCCAA*CTTTTGTTCCCTTTAGTGAG* |
| Xp21.1 | OTC | NM_000531 | *CCAGTGAATTGTAATACGAC*CACCGATCTCGAACTACCCAGTTCTTCAGTAACAGAATGAGTTGG | TTTATGGGGAAAAGAGAAGAGAATC*CTTTTGTTCCCTTTAGTGAG* |
| Xp21.1 | OTC | NM_000531 | *CCAGTGAATTGTAATACGAC*GAAAGGCAGACCAGTGCGAAATGGGCTGTCAGATTTGTACCATCC | TATCCAGATCCTGGCTGATTACCTC*CTTTTGTTCCCTTTAGTGAG* |
| Xp21.2 | DMD | AF213401 | *CCAGTGAATTGTAATACGAC*CTGCTGCTCGTCCTTGGTTTGGGATCACTCACTTTCCCCCTACAG | GACTCAGATCTGGGAGGCAATTACC*CTTTTGTTCCCTTTAGTGAG* |
| Xp21.2 | DMD | AF213406 | *CCAGTGAATTGTAATACGAC*GGAGAGAGGATGAGGGTAGTTAATCAACTTCACCACCAGCTGGTC | TGATGGCCTGGCTTTGAATGCTCTC*CTTTTGTTCCCTTTAGTGAG* |
| Xp22.2-p22.1 | RPS6KA3 | NM_004586 | *CCAGTGAATTGTAATACGAC*CAGACGTAACACGAGCAGCAATGGACAGCAAATTATGGATGAACC | TATGGGAGAGGAGGAGATTAACCCA*CTTTTGTTCCCTTTAGTGAG* |
| Xp22.2-p22.1 | RPS6KA3 | NM_004586 | *CCAGTGAATTGTAATACGAC*GACTTGAGAAGCACCGTCCAGTGACCTCAGTGAGATATTTGGTAC | CATGGTGTAAGCTGATAGCACAAGT*CTTTTGTTCCCTTTAGTGAG* |
| Xp22.2-p22.1 | PPEF1 | NM_152226 | *CCAGTGAATTGTAATACGAC*CCACCACTACCAACTCTCCACGTTCTTGTCTACCCCAATGACCTG | CACTTGAACAGAGGGAACCACGAAG*CTTTTGTTCCCTTTAGTGAG* |
| Xp22.2-p22.1 | PPEF1 | NM_152226 | *CCAGTGAATTGTAATACGAC*GGTGCAGGAATGGTGGAGAAGAATCTATGACTGAAGAGGATCGGC | TAAGAGTGGTTCCTCGCAGCTTAAA*CTTTTGTTCCCTTTAGTGAG* |
| Xp22.2-p22.13 | GRPR | NM_005314 | *CCAGTGAATTGTAATACGAC*CCCTCCAACACTCTCCTACAACTGTTTCCTTCTGAACTTGGAGGT | GGACCATTTCATGCACTGCAACATC*CTTTTGTTCCCTTTAGTGAG* |
| Xp22.2-p22.13 | GRPR | NM_005314 | *CCAGTGAATTGTAATACGAC*GACGAGGAAAGCGACCAACATACCTGCTGAGCAAGAGTTTCAGGA | AACAGTTCAACACTCAGCTGCTCTG*CTTTTGTTCCCTTTAGTGAG* |
| Xq22.3-q23 | PAK3 | NM_002578 | *CCAGTGAATTGTAATACGAC*GTCCATGAGGTGCTTGGCTTTCCACTGAGGATGAATAGTAACAACC | GGGATTCTTCAGCACTCAACCACAG*CTTTTGTTCCCTTTAGTGAG* |
| Xq22.3-q23 | PAK3 | NM_002578 | *CCAGTGAATTGTAATACGAC*CTCCACCCTCCACTCACATTCCTCTGCTGAAAATGCCAATTCCAG | TACTTTGTACAGGAACACAGATCGG*CTTTTGTTCCCTTTAGTGAG* |
| Xq22.3-q23 | FACL4 | AB061713 | *CCAGTGAATTGTAATACGAC*GGGTGAGTTGGGTAGGAAAGAGCCCACTTCAGACAAACCTGGAAG | TCCATATCGCTCTGTCACACACTTC*CTTTTGTTCCCTTTAGTGAG* |
| Xq22.3-q23 | FACL4 | AB061713 | *CCAGTGAATTGTAATACGAC*CTTGGCGTTGTCTTCGTGTCGTCTTATTGACAGTTGTGCAGGAGG | TAGCCTGGTGGTTTTCAACCTCTAG*CTTTTGTTCCCTTTAGTGAG* |
| Xq25-q26 | PDCD8 | DQ016496 | *CCAGTGAATTGTAATACGAC*GGACAAACGAACCAGCAACGAAGGAAATATGGGAAAGATCCTCCC | CGAATACCTCAGCAACTGGACCATG*CTTTTGTTCCCTTTAGTGAG* |
| Xq25-q26 | PDCD8 | DQ016496 | *CCAGTGAATTGTAATACGAC*GGTAGGGTTGAGGACGATAGGATCATTAAGGACGGTGAGCAGCAT | GAAGATCTCAATGAAGTAGCCAAAC*CTTTTGTTCCCTTTAGTGAG* |
| Xq28 | DKC1 | NM_001363 | *CCAGTGAATTGTAATACGAC*GTCCCTGGTGCTGTCTCTTTTTGGTTTCTGAGTAGTGAAGGCCAC | TTGAAGCTGGAGGAGAAACTAAAGC*CTTTTGTTCCCTTTAGTGAG* |
| Xq28 | DKC1 | NM_001363 | *CCAGTGAATTGTAATACGAC*CTCGCTTCCTCCGTCTTCACATAAGAAGAAAAAGGAGCGGAAG | TCATTGCCAGAAGAAGATGTAGCCG*CTTTTGTTCCCTTTAGTGAG* |
| Xq28 | MECP2 | NM_004992 | *CCAGTGAATTGTAATACGAC*GCTACCTGGCAACATAGGCAGCGCAAAGACATTGTTTCATCCTCC | ATGCCAAGGCCAAACAGAGAGGAGCCTG*CTTTTGTTCCCTTTAGTGAG* |
| Xq28 | MECP2 | NM_004992 | *CCAGTGAATTGTAATACGAC*CCGACCACCAGAGAAAGCAATCTGTAGACCAGCTCCAACAGGATT | CCATGGTAGCTGGGATGTTAGGGCT*CTTTTGTTCCCTTTAGTGAG* |
| Yq11.221 | CDY2A | NM_004825 | *CCAGTGAATTGTAATACGAC*CCTTCTTTGCTGGGTTTGCGCTGTTCCAATCCCGTGTCTTTCCTC | ATGGCTTCCCAGGAGTTTGAGGTTG*CTTTTGTTCCCTTTAGTGAG* |
| Yq11.221 | CDY2A | NM_004825 | *CCAGTGAATTGTAATACGAC*GGGATGGAGGAAGGAGTTAGGAGGTGCTGAGGAAGATCTGGAGCT | CAGCCCAAGGGATAGAATCCATGTT*CTTTTGTTCCCTTTAGTGAG* |
| Yq11.23 | YRRM1 | X76059 | *CCAGTGAATTGTAATACGAC*CCACTCTCACCTACCATCTCGCGCACGACAATGGTAGAAGCAGAT | CATCCTGGCAAGCTTTTCATTGGTGG*CTTTTGTTCCCTTTAGTGAG* |
| Yq11.23 | YRRM1 | X76059 | *CCAGTGAATTGTAATACGAC*CCCACTTCCCATCTACACTCGGTAGCTCAAGTTATGTGGCATCTA | TAGTAGATGGTGGGGAGAGTCGATC*CTTTTGTTCCCTTTAGTGAG* |
| Yq11.223 | PRY | NM_004676 | *CCAGTGAATTGTAATACGAC*GACTCACCCTACCTGCTATCCCCAAGAGACCACTCAAGGACAATG | GGAGCCACTGGGCTTGGCTTTCTAC*CTTTTGTTCCCTTTAGTGAG* |
| Yq11.223 | PRY | NM_004676 | *CCAGTGAATTGTAATACGAC*CGTGTTCTCGTGTTTGTGGCGGACATCTGTGGCCACGTTCATGAA | GAAGACTAAGCCTACTTCATCTCAG*CTTTTGTTCCCTTTAGTGAG* |
| Yp11.2 | TSPY2 | NM_022573 | *CCAGTGAATTGTAATACGAC*GGGAAGAGAGGTGGAAGAGTCCACTCCAATTGAGTGGTATCCGGA | TTATGAAGTGGAGGCCTATCGCCGC*CTTTTGTTCCCTTTAGTGAG* |
| Yp11.2 | TSPY2 | NM_022573 | *CCAGTGAATTGTAATACGAC*CATCCCTACACGTCCTCGATAGTTACAGCCATATGCAGGACGGCA | GTACTCAGCATGGTCTTATGCACAG*CTTTTGTTCCCTTTAGTGAG* |
| Yp11.3 | SRY | NM_003140 | *CCAGTGAATTGTAATACGAC*GCTGTTGCGTGTGCTCTTCTCTCTCTGTGCATGGCCTGTAATTT | CTGTGCCTCCTGGAAGAATGG*CTTTTGTTCCCTTTAGTGAG* |
| Yp11.3 | SRY | NM_003140 | *CCAGTGAATTGTAATACGAC*CTCAGGTCGTTGTTGCTCTGCGGCTTCAGTAAGCATTTTCCAC | TGGTATCCCAGCTGCTTGCTGAT*CTTTTGTTCCCTTTAGTGAG* |
