# Supplementary figures and images for: Rapid screening for chromosomal aneuploidies using array-MLPA
Source: BMC Med Genet. 2011 May 17;12:68. doi: 10.1186/1471-2350-12-68 (PMC3111339; doi:10.1186/1471-2350-12-68)

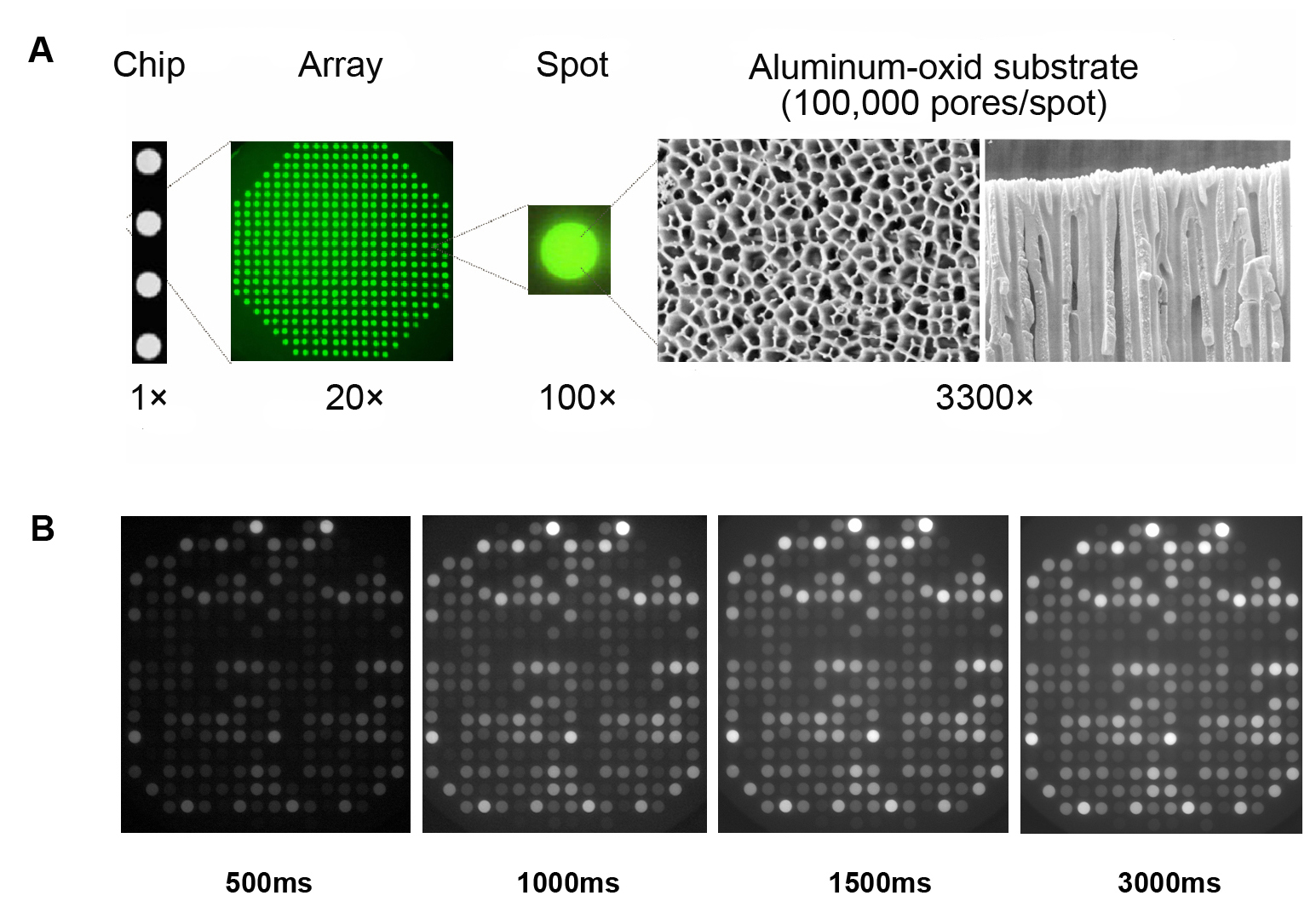

Supplement: Additional file 2 — Figure S1: Schematic depiction of the array technology. (A) Four-well array system was used in the study. Each array contained 412 spots, and the oligonucleotide was immobilized on an aluminum-oxide substrate. The substrate had a thickness of 60 μm with long capillary pores. The diameter of an individual pore was between 100 and 200 nm. A single spot occupied about 100,000 pores of the substrate. (B) Raw image obtained on an array. These images were recorded at 500 ms, 1000 ms, 1500 ms and 3000 ms exposure time using a Cy5 filter set. This array design contained 120 probes spotted in duplicate (240 spots), including 116 universal tag-probe oligonucleotides for different chromosomes, two exogenous target ArrayControl RNA Spikes (AM1780, Ambion, Austin, TX) oligonucleotides used as a negative control for array hybridization, and two Cy5-reference oligonucleotides. [file 1471-2350-12-68-S2.JPEG]
