# Supplementary material for: Rapid screening for chromosomal aneuploidies using array-MLPA
Source: BMC Med Genet. 2011 May 17;12:68. doi: 10.1186/1471-2350-12-68 (PMC3111339; doi:10.1186/1471-2350-12-68)
Supplement: Additional file 3 — Table S2: Gene copy numbers on array-MLPA in normal controls [file 1471-2350-12-68-S3.DOC]

**Table S2. Gene copy numbers on array-MLPA in normal controls**

| **Probe name** | **MLPA product size (bp)** | **Relative probe signal** | | |
| --- | --- | --- | --- | --- |
|  |  | **Normal male** | **Normal female** |  |
| 5-IL4 | 107 | 0.86 | 1.17 |  |
| 11-LMO2 | 110 | 1.03 | 1.23 |  |
| 12-IFNG | 110 | 1.25 | 0.76 |  |
| 15-HERC1 | 112 | 1.12 | 0.96 |  |
| 13-BRCA2 | 110 | 0.99 | 1.12 |  |
| 13-CCNA1 | 110 | 1.06 | ***1.22*** |  |
| 13-SMAD9 | 110 | 0.95 | 1.07 |  |
| 13-DCAMKL1 | 110 | 1.07 | 1.08 |  |
| 13-RB1 | 110 | 1.06 | 1.13 |  |
| 13-DLEU1 | 110 | 1.08 | 1.05 |  |
| 13-DLEU1 | 110 | 1.07 | 0.95 |  |
| 13-ABCC4 | 110 | 1.07 | 0.99 |  |
| 13-ING1 | 110 | 1.00 | 0.93 |  |
| 13-P85SPR | 110 | 1.06 | 0.88 |  |
| 13-P85SPR | 110 | 1.14 | 0.93 |  |
| 18-CIDEA | 110 | 0.97 | 1.05 |  |
| 18-CIDEA | 110 | 1.11 | 1.05 |  |
| 18-TWSG1 | 110 | 1.10 | ***1.56*** |  |
| 18-TYMS | 110 | 1.02 | 1.14 |  |
| 18-TYMS | 110 | 1.03 | ***1.30*** |  |
| 18-SS18 | 110 | 0.93 | 1.04 |  |
| 18-MOCOS | 110 | 0.94 | 1.01 |  |
| 18-MOCOS | 110 | 1.10 | ***1.23*** |  |
| 18-PMAIP1 | 110 | 0.92 | 1.01 |  |
| 18-PMAIP1 | 111 | 0.91 | 1.06 |  |
| 18-MBP | 110 | 1.02 | 0.97 |  |
| 21-USP25 | 110 | 0.94 | 0.95 |  |
| 21-NCAM2 | 110 | 1.09 | 0.97 |  |
| 21-APP | 107 | 1.08 | 0.98 |  |
| 21-APP | 110 | 0.98 | 0.96 |  |
| 21-TIAM1 | 110 | 0.90 | 0.88 |  |
| 21-SOD1 | 110 | 1.12 | ***1.18*** |  |
| 21-SIM2 | 110 | 0.95 | ***1.27*** |  |
| 21-TFF1 | 110 | 1.00 | 1.02 |  |
| X-OTC | 110 | 0.53 | 0.92 |  |
| X-RPS6KA3 | 110 | 0.53 | 1.02 |  |
| X-RPS6KA3 | 110 | 0.49 | 1.09 |  |
| X-PPEF1 | 110 | 0.51 | 0.89 |  |
| X-GRPR | 110 | ***0.93*** | ***0.74*** |  |
| X-PAK3 | 111 | 0.44 | ***0.81*** |  |
| X-FACL4 | 110 | 0.48 | ***1.17*** |  |
| X-FACL4 | 110 | 0.46 | 1.06 |  |
| X-MECP2 | 110 | 0.54 | 0.93 |  |
| Y-RBMY | 111 | 0.49 | 0.04 |  |
| Y-PRY | 110 | 0.50 | 0.01 |  |
| Y-PRY | 110 | 0.46 | 0.06 |  |
| Y-TSPY | 110 | 0.48 | 0.00 |  |

Note: the relative probe signals which were beyond the region from 0.85 to 1.15 were shown in italic bold.
